# Invasive tracheal aspergillosis after chemoradiotherapy treatment

**DOI:** 10.1002/rcr2.473

**Published:** 2019-08-07

**Authors:** Shok Yin Lee, Phan Nguyen, Sally Chapman

**Affiliations:** ^1^ Department of Thoracic Medicine Royal Adelaide Hospital Adelaide South Australia Australia; ^2^ Adelaide Medical School, Faculty of Health and Medical Sciences The University of Adelaide Adelaide South Australia Australia

**Keywords:** Bronchoscopy and interventional techniques, infection and inflammation, pathology, respiratory infections (non‐tuberculous)

## Abstract

Invasive tracheobronchial aspergillosis is a rare disease with most reported cases in the literature occurring in immunocompromised hosts. We report an unusual case of a patient with persistent cough and dyspnoea in the context of prior chemoradiotherapy for primary lung cancer. Chest computed tomography (CT) demonstrated an abnormal soft tissue mass surrounding the trachea and carina, with focal moderate fluorodeoxyglucose (FDG) activity on positron emission tomography (PET) scan. Bronchoscopic biopsy revealed fungal hyphae associated with necrosis and cartilage invasion, subsequently confirmed to be Aspergillus fumigatus complex. The patient was commenced on antifungal therapy promptly and had a good clinical response to treatment.

## Introduction

Invasive tracheobronchial aspergillosis is a form of invasive pulmonary aspergillosis (IPA) predominantly confined to the tracheobronchial tree. It is rare and most commonly occurs in immunocompromised hosts. The incubation period is unclear. It results in a high mortality rate if not treated. We present an unusual case of invasive tracheobronchial aspergillosis in the context of prior chemoradiotherapy treatment for lung carcinoma.

## Case Report

A 54‐year‐old female with a history of Stage IIIA primary lung adenocarcinoma which was treated with combined chemoradiotherapy followed by right middle lobectomy for single station N2 nodal disease described six months’ history of productive cough, dyspnoea, and hoarseness of voice starting two months after the completion of chemoradiotherapy with intermittent course of steroids. Her other medical history includes ductal carcinoma in situ treated with double mastectomy, asthma, and Factor V Leiden mutation with previous pulmonary embolism. She was not immunosuppressed with normal leukocyte and neutrophil count. She was on Fluticasone propionate/Salmeterol for her asthma.

**Figure 1:**
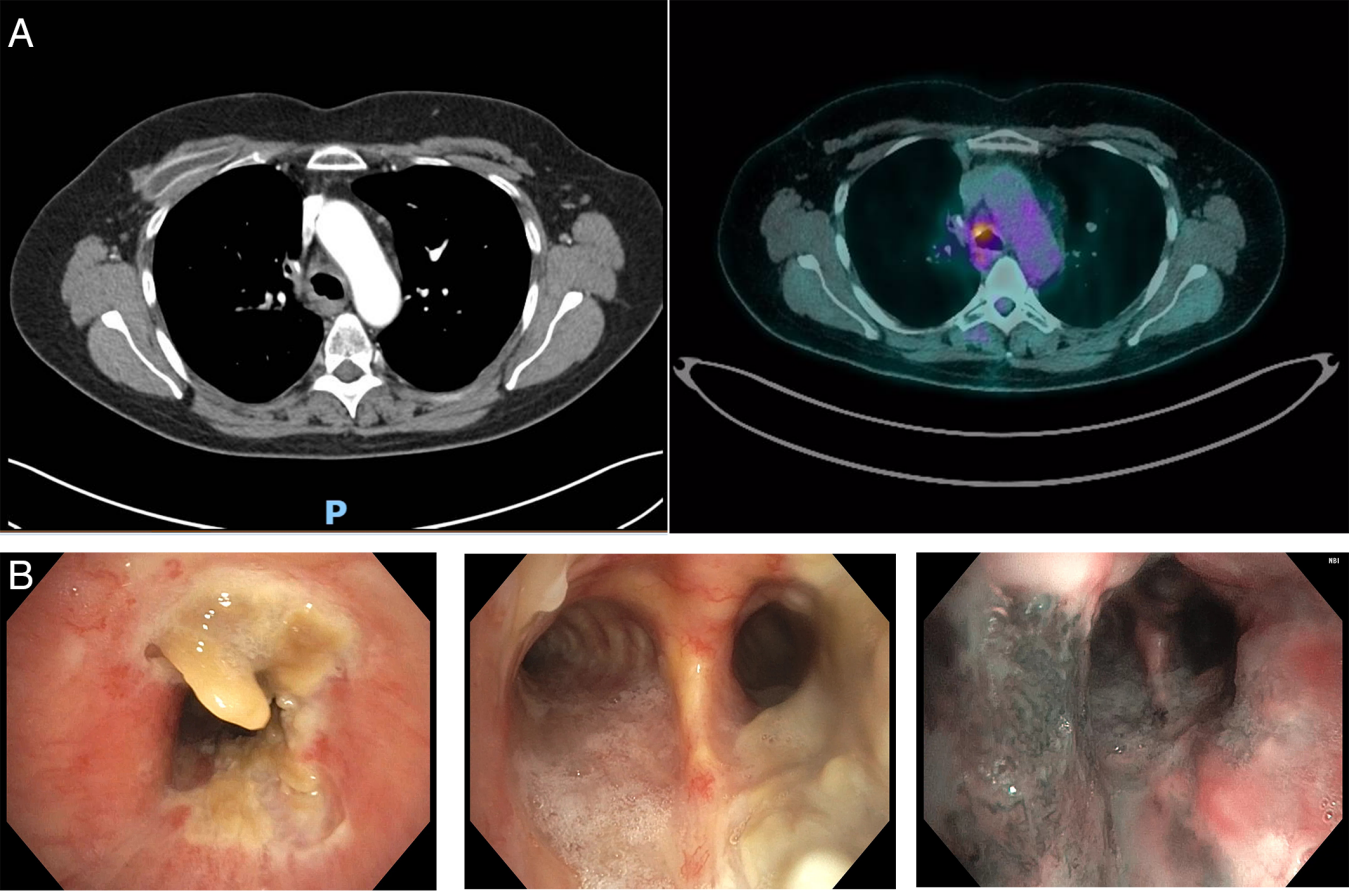
(A) Computed tomography (CT) chest and 18‐fluorodeoxyglucose–positron emission tomography/CT of distal trachea near carina, (B) trachea and distal carina pre‐treatment under white light and narrow band imaging.

**Figure 2:**
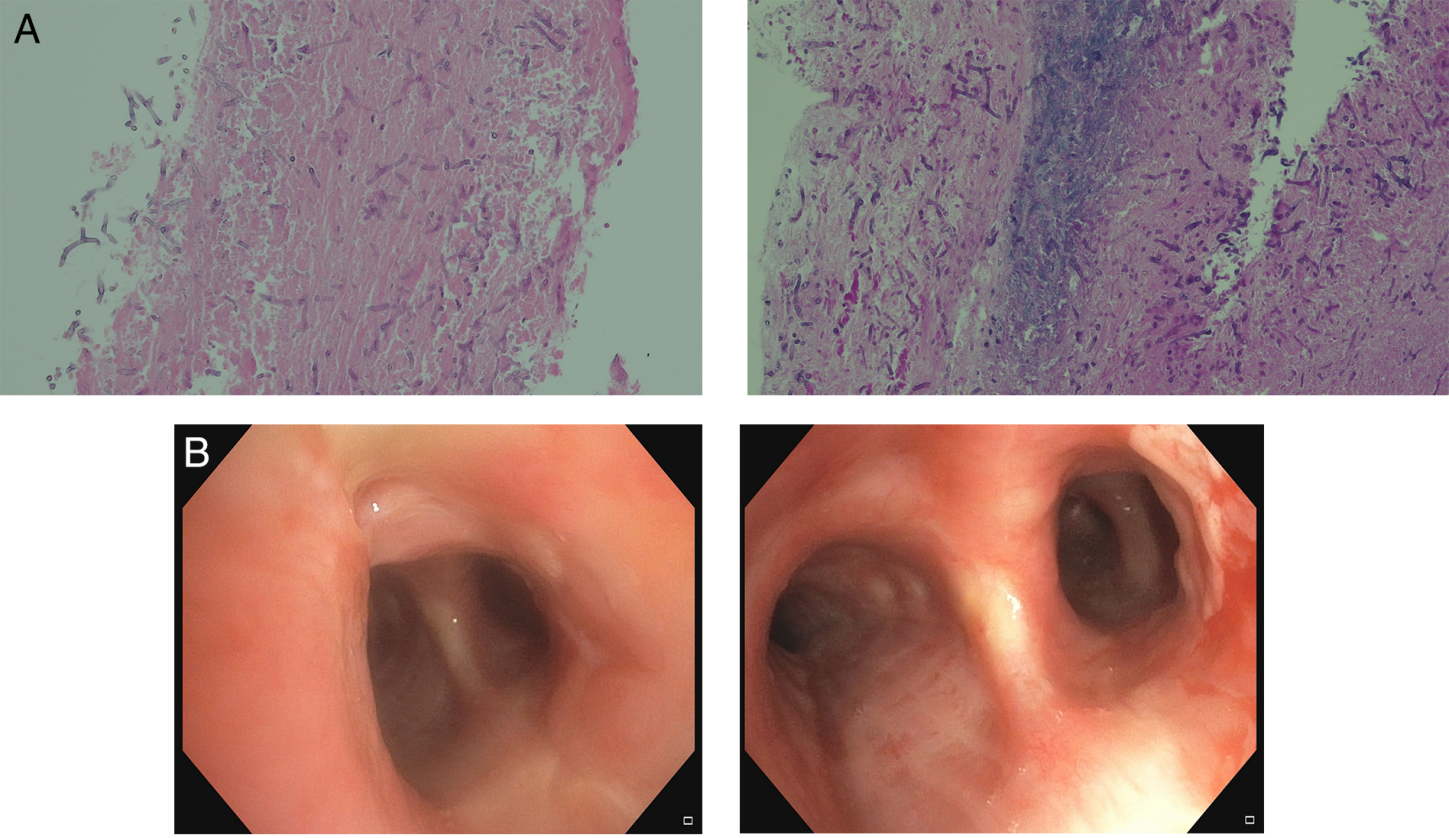
(A) Fungal hyphae under low and high power views and (B) trachea and distal carina post voriconazole treatment.

Chest computed tomography (CT) showed an abnormal soft tissue mass surrounding the trachea and carina extending to the main bronchi with associated tracheal narrowing and nodular irregularity of the contour on the right lateral border. This latter area had moderate fluorodeoxyglucose activity (SUV maximum of 4.9) on positron emission tomography scan, raising the possibility of fibrosing mediastinitis secondary to radiotherapy (Fig. 1A). Bronchoscopic evaluation showed tracheal narrowing with ulceration and necrotic tissue at the distal trachea and proximal right main bronchus (Fig. 1B). The mucosal surface looked inflamed under white light and narrow band imaging (NBI), with the left lateral wall of trachea showing particular hypervascularity (Fig. 1B). Mucosal biopsies showed abundant fungal hyphae associated with necrosis and cartilage invasion consistent with *Aspergillus* sp. (Fig. 2A). Tissue culture identified *Aspergillus fumigatus complex*, sensitive to voriconazole, itraconazole, and amphotericin B. Galactomannan test was positive.

She was commenced on oral voriconazole with significant symptomatic improvement. Repeat bronchoscopic assessment confirmed improvement in proximal airways after approximately two months of therapy (Fig. 2B).

## Discussion

*Aspergillus* is a genus of moulds 1. *Aspergillus fumigatus*, which mainly originates from decomposing vegetable material, is the most rapidly growing *Aspergillus* sp. 2. The microscopic spores enables *Aspergillus* sp. to penetrate the lungs effectively. *Aspergillus fumigatus* binds to laminin and fibrinogen more efficiently than other *Aspergillus* sp., enabling it to adhere to the airways better 2. The clinical syndromes vary from aspergilloma to severe fulminant IPA. Risk factors include pre‐existing pulmonary disease such as emphysema and pulmonary infarction, comorbidities such as diabetes, and immunocompromised hosts with solid organ or bone marrow transplantations or HIV/AIDS 1.

*Aspergillus* tracheobronchial involvement is mainly described in immunosuppressed individuals. It is most commonly seen in solid organ transplant, haematological malignancies, and patients with acquired immunodeficiency syndrome 3. Classifications of IPA include *Aspergillus* bronchopneumonia, acute tracheobronchitis (ulcerative and pseudomembranous tracheobronchitis) and obstructing bronchopulmonary aspergillosis (OBA) 4.

*Aspergillus* bronchopneumonia results in peribronchial regions of consolidation and *Aspergillus* organisms are seen deep to the basement membrane of the airway and immediately adjacent pulmonary parenchyma on histology 4. Acute tracheobronchitis is usually restricted to larger airways and has characteristic findings of airway ulceration or “plaque‐like” inflammatory lesions associated with pseudomembranous changes on bronchial walls bronchoscopically. Histologically, there is ulceration on the surface of the airway epithelium with intense submucosal inflammation. CT findings are usually normal with occasional tracheal or bronchial wall thickening 4. OBA is characterized by endobronchial involvement of *Aspergillus* sp., leading to symptoms of wheeze or stridor. CT findings are similar to that of allergic bronchopulmonary aspergillosis (ABPA) with airway dilatation, large mucoid impactions mainly involving lower lobes and diffuse lower lobe consolidation 4. In this case, our patient had ulcerative *Aspergillus* tracheobronchitis as confirmed on histology.

The most common symptoms of IPA are cough, dyspnoea, haemoptysis, and fever. There may be audible wheeze or stridor and respiratory distress resulting from significant airway narrowing which may require urgent intervention 1. Chest CT is recommended if clinical suspicion for IPA is raised 5. Leucocytes and plasma chemistry are usually normal 2. This is consistent with this case in which she presented with cough and non‐specific chest pain with normal leukocyte count.

Mortality from invasive aspergillosis is reported to be nearly 100% if not treated and the rate of progression varies. Although proven hyphal invasion on histopathology is the current gold standard of diagnosis, a culture positive for *Aspergillus* and characteristic radiological features are adequate for commencement of antifungal therapy. Surgery is an option for localized disease and significant haemoptysis 2. Voriconazole is the recommended first line therapy according to Infectious Diseases Society of America (IDSA), followed by amphotericin B and isavuconazole. Prompt treatment is indicated in patients with high suspicion of IPA while awaiting investigation results 5. Voriconazole was shown to have better treatment response and higher survival rate in comparison to amphotericin B 2. IDSA has recommended that IPA be treated with antifungal therapy for at least six to 12 weeks, based on the level of immunosuppression, regions involved and clinical response to therapy 5.

This case is an unexpected presentation of invasive tracheobronchial aspergillosis in a relatively immunocompetent host. Due to an unpredictable incubation period and variable symptoms prior to presentation, diagnosis can sometimes be delayed with a high mortality rate if not promptly treated. In her situation, cancer recurrence and radiation mediastinitis were first considerations due to her background history. Subsequent biopsy of the affected area confirmed the correct diagnosis allowing directed therapy with a good clinical outcome.

## Disclosure Statement

Appropriate written informed consent was obtained for publication of this case report and accompanying images.
